# Proteomics data repositories: Providing a safe haven for your data and acting as a springboard for further research

**DOI:** 10.1016/j.jprot.2010.06.008

**Published:** 2010-10-10

**Authors:** Juan Antonio Vizcaíno, Joseph M. Foster, Lennart Martens

**Affiliations:** a EMBL Outstation, European Bioinformatics Institute, Wellcome Trust Genome Campus, Hinxton, Cambridge, UK; b Department of Medical Protein Research, VIB, B-9000 Ghent, Belgium; c Department of Biochemistry, Ghent University, B-9000 Ghent, Belgium

**Keywords:** CV, Controlled Vocabulary, HGNC, HUGO Gene Nomenclature Committee, MCP, Molecular and Cellular Proteomics, MRM, Multiple Reaction Monitoring, NIH, National Institutes of Health, OLS, Ontology Lookup Service, PICR, Protein Identifier Cross-Referencing, PSI, Proteomics Standards Initiative, QC, Quality Control, SRM, Selected Reaction Monitoring, SBEAMS, Systems Biology Experiment Analysis Management System, TPP, Trans Proteomics Pipeline., Proteomics, Databases, Bioinformatics, Data standards, Repositories

## Abstract

Despite the fact that data deposition is not a generalised fact yet in the field of proteomics, several mass spectrometry (MS) based proteomics repositories are publicly available for the scientific community. The main existing resources are: the Global Proteome Machine Database (GPMDB), PeptideAtlas, the PRoteomics IDEntifications database (PRIDE), Tranche, and NCBI Peptidome. In this review the capabilities of each of these will be described, paying special attention to four key properties: data types stored, applicable data submission strategies, supported formats, and available data mining and visualization tools. Additionally, the data contents from model organisms will be enumerated for each resource. There are other valuable smaller and/or more specialized repositories but they will not be covered in this review. Finally, the concept behind the ProteomeXchange consortium, a collaborative effort among the main resources in the field, will be introduced.

## Graphical Abstract

**Figure:**
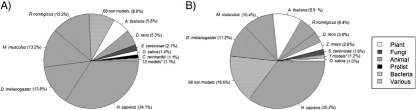

## 1 Introduction

Public availability of data has been of paramount importance in the fast development of most of the life sciences, and has become one of the foundations of modern biology. Indeed, researchers can now freely access DNA sequence information [1], microarray and gene expression data [2,3], and small molecules and chemicals [4]. At the protein level, well-annotated protein sequences can be accessed in UniProt [5], protein structures in the Protein Data Bank (PDB) [6], protein modifications in UniMod [7] and RESID [8], and protein interactions in the various resources forming the IMEX consortium [9].

This public availability of data is particularly interesting for model organisms, as they have been most vigorously researched using various high-throughput ‘omics’ analytical methodologies over the last two decades. Indeed, a large proportion of the wealth of data thus obtained has become publicly available to the research community *via* various resources, including the ones mentioned above. While the field of MS proteomics is therefore currently ahead of certain other “omics” approaches (e.g. metabolomics and glycomics) in terms of public data availability, it is still trailing other well-established “omics” disciplines such as genomics and transcriptomics in this respect. Indeed, compared to these more mature fields, relatively few MS proteomics data are currently available in the public domain, despite the increasing popularity of the approach. As a consequence, mandatory full data disclosure in the field of MS proteomics remains an important work in progress [10].

This unfortunate situation is all the more regrettable since there is some biological information that is uniquely available through MS proteomics data. For instance, transcriptomics approaches cannot predict accurately changes in active, mature protein levels in a quantitative way. Indeed, several studies have shown that a simple deduction of protein concentrations from mRNA transcript analyses is not appropriate [11–13].

Another topic that can only be well studied using MS proteomics approaches is the detection and quantification of co- or post-translational protein modifications [14]. A last point to consider here is the use of proteomics as a valuable tool for clinicians to develop new diagnostic methods or to identify biomarkers. While microarray-based approaches have also been used for this purpose, their usefulness may ultimately be more limited. The main reason is that proteomics, unlike transcriptomics, also has access to secreted, circulating proteins in different proximal body fluids such as blood plasma, serum, or urine, which present highly convenient targets for detection and quantification [15,16].

Despite the absence of a universal directive to make published MS proteomics data publicly available, several suitable repositories have been established to address the demand for storage and availability of proteomics data in the public domain. In parallel to the intrinsic complexity of the field, proteomics repositories are quite heterogeneous and have different interests and focus. Clearly, this variation is one of the many reasons why data sharing in proteomics remains limited: the current situation is simply too confusing for researchers in the field. Therefore, it is important to mention here that no single proteomics data resource will be ideally suited to all possible use cases and all potential users. As a matter of fact, existing resources already display a remarkable complementarity in that respect.

The main publicly available databases for proteomics data are the Global Proteome Machine Database (GPMDB) [17], PeptideAtlas [18], the PRoteomics IDEntifications database (PRIDE) [19], Tranche (http://www.tranche.proteomecommons.org), and the most recent addition to the list, NCBI Peptidome [20]. Additionally, there are other very valuable proteomics resources such as Human Proteinpedia [21], PepSeeker [22], the Genome Annotating Pipeline (GAPP) [23], MAPU [24], OPD [25], and the Yeast Resource Center (YRC) Public Data Repository [26]. They will not be discussed here however, since they either they are either too specialized, or contain data of human origin only and not from model organisms (*e.g.* Human Proteinpedia and MAPU). Additionally, the capabilities of these other resources have already been reviewed recently [27,28]. Other proteomics resources that store spectral libraries will not be reviewed here either. If the reader is interested in such resources, the same reviews previously cited constitute a good starting point for these specialized resources [27,28].

Sharing data is generally considered to be good scientific practice, since it allows other researchers to access, validate and reanalyze one's findings. Thus, it makes possible the confirmation of original results, the identification of errors and allows the results to be used in novel ways. Additionally, global analyses (*meta*-analyses) of heterogeneous high volume data from different origins and obtained using different experimental approaches, allow the extraction of novel insights, providing added value to the original data. Such *meta*-analyses in proteomics have been performed on data from the HUPO Plasma Proteome [29] and Brain Proteome projects [30]. Furthermore, proteomics resources offer unique additional analytical opportunities for computational biologists such as research on peptide fragmentation patterns or the generation of mass spectral libraries [28].

While the current rate of data accumulation, based primarily upon voluntary submissions by authors, is already increasing, there is a clear indication in the latest trends of a much faster growth rate. This increased deposition drive is based upon incentives from journals and funding agencies alike. Several journals in the field, for instance *Proteomics* and *Molecular and Cellular Proteomics* (MCP), have been following a trend towards mandating public deposition of MS data to support the publication of related manuscripts. At the same time, several funding agencies such as the NIH in the US, and The Wellcome Trust in the UK, are also enforcing the public availability of produced data as a key requirement, with the public deposition of data envisaged as a way to maximize the value of the funds provided.

In the age of systems biology and data integration, it is furthermore essential to count on proteomics data as a crucial part of the “whole picture” of life. The aim of this review is therefore to provide an overview of the current situation regarding proteomics data repositories and to serve as a solid starting point for those who want to perform proteomics data mining in model organisms, either in a domain-specific or in an integrative way.

We will here review each of the major proteomics resources independently, although the capabilities and data contents of PRIDE will be explained with a higher level of detail. Each resource will also be analyzed in terms of the information they contain from different model organisms.

## 2 Information stored in MS based proteomics resources: data formats and content

A large variety of MS techniques are available to researchers in the field, with different experimental approaches serving different goals. As a result, some researchers will use MS to identify as many proteins as possible in their sample of interest, while others may be interested in the characterisation of just a very small subset of proteins. A rather recent development in proteomics concerns the application of targeted approaches, typically using Selected Reaction Monitoring (SRM) [31]. Furthermore, with quantification of proteins becoming increasingly important, yet more different experimental designs are being developed in the field.

This plethora of experimental approaches is relevant for data resources, since the experimental approach and the type of data generated influence the data formats that are available. In general terms, each proteomics experiment requires three main types of information to be stored. Because of the different nature of these, it is essential that all three are kept. The three data types are:

i) *Original experimental data recorded by the mass spectrometer (primary data).* The mass spectra recorded from the instrument are typically stored originally in a binary vendor or instrument specific formatted file, and data can be very hard to access for users with different experimental platforms [32]. These files can be converted to open, XML-based file formats such as mzXML [33], mzData or the new mzML format [34], that contain not only the mass spectra *per se* but also some experimental metadata. However, since these XML files can be very large and therefore difficult to handle, the most common practise remains the generation of text files containing peak lists, derived from the original instrument files [35].
ii) *Results inferred from the original primary data.* Peptide and protein sequences from sequence databases are usually inferred from the acquired mass spectra using search engines, although alternative approaches like *de-novo* sequencing and searches based on spectral libraries are becoming increasingly popular as well. Regardless of the approach however, if peptides are identified, the non-trivial job of mapping them to proteins must follow. The inherent complexity of this protein inference step is often not taken into account. Problems arise when the detected peptide sequence potentially matches several proteins. If this is the case, and it regularly is for shotgun proteomics approaches, the decision about which of the several potential proteins present in the sample is complex and controversial [36]. For this reason, the protein inference problem remains one of the bottlenecks for proteomics data analysis and storage alike.

Depending on the search engine used to obtain the data, these results can take a variety of formats, many of them are text based. However, a higher level of complexity has been introduced since experiments are increasingly being focused in the quantification of the observed proteins. The variety of methods and experimental approaches [37,38], and the fact that many of them are not mature enough, makes this kind of data very hard to be captured by the proteomics repositories. Whereas the capture of qualitative data by proteomics repositories can at present be problematic but is “under control”, the standardized incorporation of quantitative data remains very much a work in progress.

iii) *Experimental metadata.* This comprises information about author contact, sample, experimental protocol, data processing, and any other relevant piece of knowledge that gives a proper and adequate technical and biological background to the two first types of data.

Ideally, this information should be conveyed using terms from a controlled vocabulary (CV) or ontology. This way, the metadata is not only available to humans, but it is also readily computer readable and can therefore be queried easily and automatically. The alternative, using simply free text, may be readable and interpretable by humans but is impossible to query in a structured and reliable way by computers.

## 3 Proteomics data repositories and databases

The main characteristics of the existing proteomics resources are summarized in Table 1. For each of the listed resources we will divide the information into four categories: General information, data submission and format support, data mining and visualization, and data content for model organisms.

### 3.1 PRoteomics IDEntifications database (PRIDE)

#### 3.1.1 General information

The PRIDE database (http://www.ebi.ac.uk/pride) at the European Bioinformatics Institute (EBI, Cambridge, UK) was established as a public data repository to support the publication of MS related studies. Thus, PRIDE stores three different kinds of information: peptide and protein identifications derived from MS or MS/MS experiments, MS and MS/MS mass spectra as peak lists, and all associated metadata [39].

Data in PRIDE is not reprocessed or altered in any way after submission, since PRIDE represents the submitter's view of the data. PRIDE allows data to remain private while anonymously sharing it with journal editors and reviewers and it is now a recommended submission point for several journals such as *Nature Biotechnology* [40], *Nature Methods* [41], MCP and *Proteomics*.

PRIDE relies on two additional core tools: the Ontology Lookup Service (OLS) [42] and the Protein Identifier Cross-Referencing (PICR) service [43]. PRIDE uses OLS to store, structure, and present any and all metadata annotations. The widespread and persistent use of CVs and ontologies, along with the possibility to perform queries based on these annotations, present a unique feature of PRIDE when compared with any other proteomics repository. This ready availability of rich metadata in the PRIDE database has been crucial in supporting the global *meta*-analysis of large, collaborative proteomics projects [29].

The PICR tool is used to keep up-to-date mappings of all the submitted protein identifications in PRIDE to all known accession numbers (including older accession numbers that are no longer in use) for those proteins across the most important protein databases. This way, protein identifications submitted to PRIDE that were originally derived from different databases, or from different time points of the same database, become fully comparable.

#### 3.1.2 Data submission and format support

Submissions to PRIDE are performed using a publicly available XML data format called PRIDE XML. The development of the PRIDE Converter submission tool [44] (http://www.pride-converter.googlecode.com) has been key in the large growth of data content in PRIDE during the last year [39]. PRIDE Converter has made submission to PRIDE a simple and efficient process since a submitter can now convert a wide variety of the most common proteomics data formats directly to PRIDE XML in eight easy steps in a user-friendly, wizard-like graphical user interface (GUI), making the submission process much easier and more straightforward. Examples of supported formats include Mascot .dat and .mgf files [45], SEQUEST result (.out) and .dta files [46], X!Tandem .xml files [47], OMSSA .omx files [48], SpectrumMill result (.spo) and .pkl files, ProteinProphet/PeptideProphet prot.xml and pep.xml files [49], mzXML [33], MS2 [50] and mzData. At the moment PRIDE Converter handles spectrum fragmentation annotation for certain formats: Mascot .dat files, OMSSA .omx files and files from the open source LIMS system ms_lims [51]. Once the files are converted, they are automatically validated, making it possible simply to upload the resulting PRIDE XML files to an EBI FTP server [52].

Very importantly, PRIDE has always committed to using community data standards as formulated by the HUPO Proteomics Standards Initiative (PSI, http://www.psidev.info). The current PRIDE XML is therefore based on the mzData format developed by HUPO PSI [53]. In the near future, PRIDE will adopt the new formats mzML (for MS) [34] through the jmzML API [54], and mzIdentML (for peptide and protein identifications). However, at present, quantitative data in PRIDE is only supported to a limited extent [55]. The first datasets containing actual protein expression values coming from TMT/iTRAQ based approaches have been submitted, but are not yet publicly available at the time of writing.

#### 3.1.3 Data mining and visualization

Detailed, step-by-step usage of the PRIDE web interface has been described extensively before [52,56,57]. All data contained in PRIDE from a single species can be obtained *via* the PRIDE ‘Browse’ page by clicking the relevant NEWT taxonomy term.

Additionally, the PRIDE BioMart interface offers easy access to species data. BioMart usage does not require any programming skills and presents a query-oriented data management system that allows for very powerful data retrieval [58]. A link to the PRIDE BioMart is provided in the PRIDE main web page. Alternatively, PRIDE can be also queried at the current BioMart Central Portal [59] (http://www.biomart.org/biomart/martview/), where it is also used to integrate information from PRIDE with other very popular resources such as Ensembl [60], UniProt [5], Reactome [61], InterPro [62], the Macromolecular Structure Database (MSD) [63], or the IPI database [64] among a growing list of others. At the moment, it is only possible to integrate two resources at the same time but it is expected that this limitation will disappear later this year when the new version of BioMart is released.

For more sophisticated data mining purposes, users can also download the corresponding XML files for each public experiment from the EBI FTP server (ftp://ftp.ebi.ac.uk/pub/databases/pride).

At present, PRIDE relies on the submitters in terms of the quality of the existing datasets, as detailed proteomics data cannot be curated from existing literature. However, this situation has started to change since a key ongoing development is the creation of a new database called PRIDE-Q (for ‘Q-rated’) that will contain only the highest-quality data from the PRIDE repository [39].

#### 3.1.4 Data content for model organisms

**Fig. 1:**
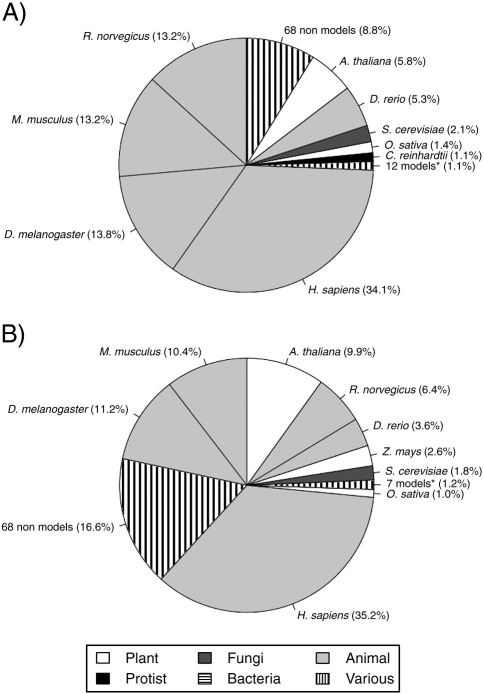
(A) A pie chart representing the protein identification contributions of model organisms in PRIDE. * The asterisk represents 12 species: *G. gallus*, *Zea mays*, *Synechocystis* sp., *C. elegans*, *E. coli*, *Xenopus laevis*, *Emiliania huxleyi*, *Macaca mulatta*, *N. tabacum*, *Ustilago maydis*, *Ashbya gossypii* and *Neurospora crassa*. (B) A pie chart representing the peptide contributions of model organisms in PRIDE. * The asterisk represents 7 species: *Synechocystis* sp., *G. gallus*, *E. coli*, *C. elegans*, *M. mulatta*, *E. huxleyi* and *N. tabacum.*

PRIDE contains a diverse range of organisms from all kingdoms reflecting the varied interests and applications of proteomics for studying fundamental biological phenomena. However, it is the model organisms that are most useful in probing the complexities of common across-species traits. At the start of February 2010, PRIDE contained about 2.9 million protein identifications and 13.3 million peptide identifications, with an average of 5.6 spectra *per* peptide identification. Twenty-one model organisms are represented, ranging from *Synechocystis* species (a cyanobacteria used in photosynthetic research) to *D. rerio* (zebra fish), a common model for development and toxicology studies. Based on the number of protein identifications, *Homo sapiens* is the most represented species in PRIDE, with over twice as many identifications as the next most common set of model organisms: *Drosophila melanogaster*, mouse and *Arabidopsis thaliana* (Fig. 1A).

**Fig. 2:**
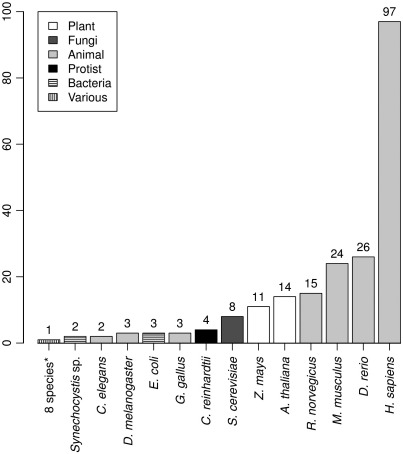
Model organism representation by number of data depositions in PRIDE. * The asterisk represents 8 species: *X. laevis*, *U. maydis*, *O. sativa*, *N. tabacum*, *N. crassa*, *M. mulatta*, *E. huxlei* and *A. gossypii*.

The peptide level shows similar statistics, with *H. sapiens* having more than twice the peptide identifications assigned than the next nearest model organism *D. melanogaster* (Fig. 1B). It is interesting to note that when kingdoms are compared, there is large bias towards animals, with plants coming a distant second in both peptide and protein identifications. Data about the number of data depositions (groups of experiments submitted at different time points, Fig. 2) show that, as expected, *H. sapiens* comes with the largest number of distinct data depositions. Animal model organisms such as zebra fish, mouse and rat are all well represented (26, 24 and 15 data depositions respectively). Figures for plants (*Arabidopsis* and maize) are a bit lower (14 and 11 data depositions, respectively), and the numbers of distinct data submissions for some key model organisms such as *Caenorhabditis* *elegans* and *D. melanogaster* remain only sporadic.

From PRIDE, a researcher can get the original author's view on the data (peptide and protein identifications, and mass spectra as peak lists) coming from a wide variety of organisms, since data are not reprocessed in any way. Thanks to the extensive use of controlled vocabularies to report metadata, it is possible to query PRIDE using a large variety of criteria, and the proteomics information can be linked to other types of biological data through various highly popular external resources (such as Ensembl or UniProt, amongst others) using the BioMart. An up to date view on the protein identifications reported is always available thanks to the continuous use of the PICR service. Targeted approaches are not supported at the moment and no information is provided about proteotypic peptides.

### 3.2 The Global Proteome Machine database (GPMDB)

#### 3.2.1 General information

The Global Proteome Machine database (http://www.thegpm.org/) [17] was developed by Beavis informatics. MS/MS data is (re-)processed mainly using the popular open source search engine X!Tandem [47], although other related tools are now available as well. Peptide and protein identifications are generated and stored in the GPMDB. One of the ways to access the data is through the boutique proteomes, which are a collection of species-specific databases. Users can then restrict the peptide identification analysis to a specific species by choosing to search a particular proteome.

#### 3.2.2 Data submission and format support

MS/MS data in different formats (.dta, .pkl, .mgf, mzXML, and mzData) can be submitted to the GPM via the “simple search page.” For files that are too big, a compressed format called Common 1.0 (.cmn) can be used. Converter tools from peak list formats to compressed .cmn files are also provided. Once the data has been processed *via* X!Tandem, users can choose whether or not to submit their data to GPMDB. Private data submissions are also allowed. GPMDB data is stored in XML files, which are indexed in a MySQL database.

In addition to X!Tandem, users can also choose to use a different search engine called X!Hunter to (re-)process their data [65]. X!Hunter compares the experimentally observed spectra with consensus mass spectra obtained from the GPMDB. At present, support is available for human, mouse, *A. thaliana* and *Saccharomyces* *cerevisiae*.

In order to improve comparability of data, GPMDB has also implemented automatic conversion of identifiers for different databases such as Ensembl, IPI, HGNC [66], NCBI genes and UniProt accession numbers. Experimental metadata present in GPMDB is essentially limited to the species information, sometimes including additional information about the tissue/organ and/or the subcellular location.

#### 3.2.3 Data mining and visualization

The GPMDB web interface can be searched based on keywords from protein or dataset descriptions. Another possible way to access the data is the “ontology” option, where data from human, mouse and yeast can be retrieved based on Gene Ontology (GO) terms associated with identified proteins. The visual representations available in the GPMDB web interface are quite diverse and powerful. There are three possible ways to view identified proteins: gene view (G), protein and observed peptide sequence view (P) and the X!Tandem view (X). It is also possible to see annotated spectra.

When searches have been done against the Ensembl database the results can be viewed as a KEGG [67] pathway, by sorting proteins into metabolic pathway categories. Other interesting developments include the web interface called “pSYT,” which allows users to access information on phosphorylation of proteins present in GPMDB, and also the SNAP web interface, which allows the visualization of peptides with mutations when mapped to Ensembl.

One important feature of GPMDB is the support for proteotypic peptides. Proteotypic peptides for GPMDB are defined as those that are more likely to be confidently observed, so in fact, those which are most often detected. These peptides need not be unique for a given protein. It is then possible to access all proteotypic peptides from human and yeast origin. This comprehensive list can be used by a search algorithm called X!Tandem P^3^, which will only use this list of frequently detected peptides to identify spectra in an attempt to improve the confidence of the results. If this algorithm is used, data can also be stored in GPMDB if desired by the submitter.

For advanced users, MS/MS files searched using the GPM pipeline are available for download at ftp://ftp.thegpm.org/data/msms. In addition, for some human proteins GPMDB now provides transition prediction functionality, which can be useful in the design of targeted approaches. The GPM-MRM tool is integrated in the GPM web-interface (http://www.wiki.thegpm.org/wiki/GPM-MRM).

GPMDB has not yet implemented a formal procedure to perform quality control (QC) of the existing data. However, peptide identifications are obtained through extensively used software such as X!Tandem and X!Hunter, where fixed thresholds are used in order to assess that the resulting data is reliable.

#### 3.2.4 Data content for model organisms

According to GPMDB official statistics (on February 2010), 56 million of the peptide observations are from human origin (about 42% of the peptide observations from a total of 132.3 million). However, model organisms are very well represented: mouse (15 million, 11%), *S. cerevisiae* (6.8 million, 5%), zebra fish (2.7 million, 2%), chicken and *C. elegans* (both around 2.5 million, 1.9%), *Drosophila* (2.0 million, 1.5%), *Arabidopsis* and rat (both around 1.3 million, 1%), and finally dog, *Xenopus* and rice (all of them containing around 100,000 peptide observations or less). There is more data from other model organisms in GPMDB that can be accessed through the web interface search functionality, but statistics are not available.

In addition, in the boutique proteomes it is possible to run the searches versus a number of genomes from different prokaryote model organisms such as *Escherichia* *coli*, *Bacillus subtilis*, *Caulobacter crescentus*, *Mycoplasma genitalium*, *Pseudomonas fluorescens*, the cyanobacterium *Synechocystis*, and *Vibrio fischeri*.

To summarize, researchers can get from GPMDB submitted data reprocessed through the X!Tandem and/or X!Hunter search engines: peptide and protein identifications and mass spectra. It is possible to query the data based on species information and, in certain cases also on subcellular location. Information is available about proteotypic peptides and targeted approaches are supported for human proteins.

### 3.3 PeptideAtlas

#### 3.3.1 General information

The PeptideAtlas project, at the Institute of Systems Biology (ISB, Seattle, USA) (http://www.peptideatlas.org/), annotates genome sequences of different organisms with peptides and proteins derived mainly from MS/MS data [18]. It is very important to highlight here that prior to storage, as it happens for GPMDB, all data in PeptideAtlas is reprocessed, in this case via the popular Trans Proteomics Pipeline (TPP) [68].

All the processed results are loaded into SBEAMS (Systems Biology Experiment Analysis Management System)-Proteomics, a proteomics analysis database built as a module under the SBEAMS framework. The identified sequences are then mapped onto their respective genome sequence, resulting into species or sample-specific ‘builds’, which represent all peptides mapped to a single reference Ensembl genome. As a matter of fact, PeptideAtlas is a peptide centric repository and aims to reprocess periodically the available data with new tools for identification and statistical validation, therefore providing an up to date vision of a particular proteome.

#### 3.3.2 Data submission and format support

Only MS/MS spectra are accepted, in either native raw, mzML or mzXML format. Detailed instructions for potential submitters are available in http://www.peptideatlas.org/upload/. To submit data the first step is to use the PeptideAtlas feedback form. FTP upload support is also available. Similarly to the other repositories, data can be kept private and will only become available when the submitter specifies it. PeptideAtlas also performs automatic conversion of identifiers for different databases such as Ensembl, IPI, RefSeq, Unigene and UniProt accession numbers. For each peptide, external links to other resources such as GPMDB or the Human Protein Atlas [69] are provided.

PeptideAtlas contains in some cases basic metadata per sample: *e.g.* the cell type, pathology-related information, or the experimental technique used. However, if it exists, this information is quite limited in most cases.

#### 3.3.3 Data mining and visualization

All protein identifications can be viewed *via* the Ensembl browser as Distributed Annotation System (DAS) tracks [70]. Unprocessed datasets (raw files), MS/MS files in mzXML format, output files from search engines and lists of identifications can be accessed at http://www.peptideatlas.org/repository.

One important feature of PeptideAtlas is the possibility of accessing proteotypic peptides. In this case, the proteotypic concept is more restricted than in GPMDB, since they are defined as peptides that can uniquely and unambiguously identify a specific protein. For each peptide in PeptideAtlas an empirical proteotypicity score is calculated. Peptides with high scores are then most likely to be better targets for SRM approaches. PeptideAtlas is defined as a resource for target selection for emerging targeted proteomics workflows [18] and currently supports targeted approaches in three ways:

- MRMAtlas (http://www.mrmatlas.com) is a compendium of targeted proteomics assays to detect and quantify yeast proteins in complex proteome digests by mass spectrometry [71]. It currently contains assays for nearly 1500 *S. cerevisiae* proteins (21% of the yeast proteome).
- TIQAM (Targeted Identification for Quantitative Analysis by MRM) [72], a desktop Java application to facilitate the selection of peptide and transitions. It consists of three applications: TIQAM-Digestor, TIQAM-PeptideAtlas and TIQAM-Viewer.
- MaRiMba [73], a new component of the TPP since July 2009. It makes use of spectral libraries to create transition lists. Libraries of consensus spectra can be retrieved directly from PeptideAtlas, or can be created using SpectraST [74], the spectral library building and searching tool from TPP.

In terms of data visualization, one of the nicest features of PeptideAtlas is the Cytoscape [75] plug-in, which allows the user to view the distinct peptides for a particular protein as a network with associated proteins. Finally, it is important to highlight that peptide and protein identifications are obtained through the popular TPP, and the resulting data are then expected to be trustworthy. However, equivalent to GPMDB, PeptideAtlas has not implemented a formal QC of the existing data yet.

#### 3.3.4 Data content for model organisms

As mentioned before PeptideAtlas builds are performed for individual organisms and important sample groups (e.g. plasma). By February 2010, there is a total of 11 builds available and five of them come from model organisms: *C. elegans* (last built on May 2008), *Drosophila* (July 2009), mouse plasma (December 2009, but by February 2010 it was not available for download yet), yeast (April 2009) and yeast MRM (also known as MRMAtlas as cited before, built on February 2008).

To summarize, researchers can get from PeptideAtlas a normalised view of the submitted data, reprocessed through the popular TPP pipeline: peptide and protein identifications and mass spectra. Within each build it is possible to link the information between every peptide and the corresponding gene in the Ensembl release. Information is available about proteotypic peptides (although it is a more restrictive concept than for GPMDB) and targeted approaches are widely supported.

### 3.4 Tranche

#### 3.4.1 General information

Tranche at the University of Michigan (https://proteomecommons.org/tranche/) is a distributed storage platform that supports sharing and dissemination of potentially very large files and proteomics related datasets. Tranche is based on an encrypted peer-to-peer system and data uploaded into Tranche is split into discrete units and split across multiple servers [28]. Tranche is a pure repository that hosts any kind of data and the concept of the system is analogous to a computer hard disk. Therefore, it is indeed the most universally applicable system. For this reason, Tranche is starting to store other kinds of data different to proteomics such as data sets from the personal genome project (https://proteomecommons.org/news.jsp?i=664).

#### 3.4.2 Data submission and format support

In order to submit data (in any format), it is necessary to request an account first. It is also possible to keep data private in the pre-publication stage and make it accessible for potential reviewers. Access to uploading or downloading data is given via a Java Web Start application. Once the application is loaded, users can browse data by project or using the hash codes assigned by Tranche to each dataset.

#### 3.4.3 Data mining and visualization

The interface is quite simple and complex queries cannot currently be performed since the annotation level is limited, something that can constitute a problem if someone wants to reuse the original data [76]. However, other repositories such as PRIDE and PeptideAtlas have begun to interface with Tranche as the mechanism to store and disseminate large files (*e.g.* binary files from the mass spectrometers and output files from search engines).

Thus, Tranche is an invaluable system to support the exchange of large proteomics related files, which at present cannot be stored anywhere else. These large files are meant either for power users or for other resources such as PeptideAtlas, to allow the reanalysis or reprocessing of large volumes of data. However, due to the nature of data present in Tranche, where virtually all kinds of files can be submitted and annotation can be minimal, no formal QC is performed.

#### 3.4.4 Data content for model organisms

At present, it is impossible to estimate accurately the amount of data from different species that Tranche contains, since, as mentioned before, the ‘Search Data’ web functionality is quite limited. More importantly, Tranche does not mandate the provision of experimental metadata when a dataset is submitted, so this information is simply not available for many datasets. As a matter of fact, by February 2010 Tranche contains 7101 data sets. Just as an example, if we look for certain keywords related with species names we get the following results: 305 datasets for human, 290 for rat, 78 for mouse, 51 for *S. cerevisiae* and 14 for *E. coli*. However, some of the retrieved results are not actual experimental datasets and, on the other hand, different figures are obtained depending on the exact term the search is based on.

From Tranche researchers can usually get the corresponding original raw data of a submitted proteomics experiment. Other kind of files could be available as well, depending on each submission, such as output files from the search engines used, excel files including the information related peptide and protein identifications, etc.

### 3.5 NCBI Peptidome

#### 3.5.1 General information

NCBI Peptidome (http://www.ncbi.nlm.nih.gov/peptidome) is the most recent of the main proteomics repositories launched [77] and for that reason is the one that contains the least amount of data at present. It is essentially a sibling repository to PRIDE, since data is not reprocessed in any way and the original view on the data by the submitter is represented. Peptidome stores lists of identified peptides and proteins, mass spectra as the supporting evidence for these identifications, and descriptive information about the biological samples, instrumentation and/or the informatics pipeline. As with all the previous resources, reviewer accounts can be created to access anonymously data in the prepublication stage.

There are two types of components in Peptidome: Samples (containing all the data related to the biological material, which is derived from one or more MS runs) and Studies (collection of samples from the same experiment).

#### 3.5.2 Data submission and format support

Very detailed submission guidelines are available at http://www.ncbi.nlm.nih.gov/peptidome/guidelines/#meta. Currently, there are four types of files required for a complete submission [20]: metadata file that describes the overall experiment, raw data files containing the MS and MS/MS data (formats accepted: mzData, mzXML, mzML, and peak lists such as .mgf, .pkl, .sqt or .dta files), output files from a peptide identification program (mascot .dat files, OMSSA .omx and pep.xml files are supported at the moment), and results summary tables that describe the submitter's view of the final processed results. Once all files are ready for submission, they can be transferred to the NCBI via FTP.

NCBI Peptidome also supports quantitative data to a certain extent [20]. Therefore, in the submission process it is possible to include a single quantification value per protein, peptide and/or spectrum. Also, metadata should be included to describe the units (if applicable) and the methodology.

Metadata available in Peptidome per average submission is quite rich. Therefore, detailed descriptions about the sample or the experimental protocol can be found per sample. However, it is unfortunate that at present, it is not possible to query the data based on most of the criteria stored.

#### 3.5.3 Data mining and visualization

The Peptidome web interface is the primary way to access the data. However, it is also possible to access the data in Peptidome in an indirect way *via* the NCBI's *Entrez* search and linking system [78].

In addition, all original data files (spectra files, identification results and supplementary data) are available for download at ftp://ftp.ncbi.nih.gov/pub/peptidome. To our knowledge, a formal QC of the data in Peptidome has not been implemented yet.

#### 3.5.4 Data content for model organisms

As stated before, due to Peptidome's recent launch it currently contains the least amount of data. By February 2010 it contained 23 public studies, ten of them from human origin. In terms of number of studies coming from model organisms, the most represented organism in Peptidome so far is *S. cerevisiae* (7 studies). Apart from that, there is data (one study each) from mouse, *E. coli* and *Ciona intestinalis*, the sea squirt, a major model for developmental biologists.

To summarize, researchers can get from NCBI Peptidome the original author's view on the data, since as it happens for PRIDE, data is not reprocessed in any way: peptide and protein identifications, and mass spectra as peak lists. Rich metadata is present but unfortunately, complex queries cannot be made at present. Information about proteotypic peptides is not available and targeted approaches are not currently supported.

## 4 Future perspectives and conclusions

The main proteomics repositories (PRIDE, NCBI Peptidome, Tranche, PeptideAtlas and GPMDB) are currently working on the implementation of a system called ProteomeXchange that will allow proteomics data sharing between all the members in a scheduled and well-structured way [79]. The capabilities of the different resources are complementary so the idea behind ProteomeXchange is to have a single point of submission for data deposition, while providing multiple points of access for data visualization and analysis.

PRIDE and NCBI Peptidome are envisioned as the initial submission points, Tranche is the storage place for large files and acts as the primary data transport hub, and PeptideAtlas and GPMDB will be notified immediately when datasets are made public in order to get full advantage of metadata present in the other resources before reprocessing all the data. Draft guidelines for submissions are available (see http://www.proteomexchange.org) and a large-scale ProteomeXchange pilot submission has already been performed [39]. In the same context, PRIDE and NCBI Peptidome, the most similar repositories, formally agreed that they will replicate and share all their public data, again ensuring that data becomes optimally visible to the scientific community. However, the actual exchange has not started yet at the time of writing.

All together, these initiatives are expected to help overcome the community's reticence about data disclosure in the field. However, it must also be taken into account that this data-sharing process is very resource-intensive and time-consuming for the data repositories.

It seems clear to us that more proteomics data from model organisms is needed in public repositories since the figures remain of a different order of magnitude compared to data from human origin. Communities devoted to particular organisms would be the first to reap benefit from increased data sharing, since it is clear that the higher the diversity of data going into proteomics repositories (in terms of sample, experimental technique, instrument or search engine), the higher the number of unique peptides sequences that are found [39].

Since the current volume of proteomics data deposition is rapidly increasing, new approaches based on the reanalysis of the data and/or new uses of the stored data will also become possible. Targeted approaches represent another promising field and researchers can already make heavy use of the proteomics resources in order to improve the experimental designs in these types of experiments.

## Figures and Tables

**Table 1:** Main characteristics of the major proteomics repositories and databases.

	PRIDE	GPMDB	PeptideAtlas	Tranche	Peptidome
Reprocessing of the data	No	Yes (X!Tandem and others)	Yes (TPP)	No	No
Editorial control	No	Yes	Yes	No	No
Level of annotation	Detailed	Very basic	Basic	Very basic	Detailed
Proteotypic peptides	No	Yes	Yes	No	No
Support for targeted approaches	No	Yes	Yes	No	No
Data submission	PRIDE Converter and FTP upload	Web interface	FTP upload	Web interface	FTP upload
Submission of raw (binary) data	No	No	Yes	Yes	No
Access to deposited data	Web interface, BioMart, web service, and direct download of files	Web interface and direct download of files	Web interface and direct download of files	Java Web Start application	Web interface and direct download of files
